# Model-based genotype-phenotype mapping used to investigate gene signatures of immune sensitivity and resistance in melanoma micrometastasis

**DOI:** 10.1038/srep24967

**Published:** 2016-04-26

**Authors:** Guido Santos, Svetoslav Nikolov, Xin Lai, Martin Eberhardt, Florian S. Dreyer, Sushmita Paul, Gerold Schuler, Julio Vera

**Affiliations:** 1 Laboratory of Systems Tumor Immunology, Friedrich-Alexander University of Erlangen-Nuremberg, Germany; 2 Department of Dermatology and Erlangen University Hospital and Faculty of Medicine, Friedrich-Alexander University of Erlangen-Nuremberg, Germany; 3 Systems Biology and Mathematical Modelling Group, University of La Laguna, Spain; 4 Institute of Mechanics, Bulgarian Academy of Science, Sofia, Bulgaria; 5 University of Transport, Sofia, Bulgaria

## Abstract

In this paper, we combine kinetic modelling and patient gene expression data analysis to elucidate biological mechanisms by which melanoma becomes resistant to the immune system and to immunotherapy. To this end, we systematically perturbed the parameters in a kinetic model and performed a mathematical analysis of their impact, thereby obtaining signatures associated with the emergence of phenotypes of melanoma immune sensitivity and resistance. Our phenotypic signatures were compared with published clinical data on pretreatment tumor gene expression in patients subjected to immunotherapy against metastatic melanoma. To this end, the differentially expressed genes were annotated with standard gene ontology terms and aggregated into metagenes. Our method sheds light on putative mechanisms by which melanoma may develop immunoresistance. Precisely, our results and the clinical data point to the existence of a signature of intermediate expression levels for genes related to antigen presentation that constitutes an intriguing resistance mechanism, whereby micrometastases are able to minimize the combined anti-tumor activity of complementary responses mediated by cytotoxic T cells and natural killer cells, respectively. Finally, we computationally explored the efficacy of cytokines used as low-dose co-adjuvants for the therapeutic anticancer vaccine to overcome tumor immunoresistance.

In many solid cancer types, the interaction between the tumor and the immune system is a key element governing critical steps in the tumor progression path^1^; its deep understanding is necessary to design efficient anticancer immunotherapies. In recent times, a number of published works suggest the use of a systemic approach combining quantitative experimental data and mathematical modeling to dissect the tumor-immune system interaction^2^,^3^. However, most of these modelling efforts focus on representing and simulating cell-to-cell processes and do not consider the intracellular networks controlling immune and tumor cells, thereby losing the chance to integrate and analyze omics data on the molecular events underlying the tumor-immunity interaction and the immune-based therapies.

The immune system is by definition multi-scale because it involves complex biochemical networks that regulate cell fate across cell boundaries^4^, and also because immune cells communicate with each other by direct contact or through secretion of local or systemic signals^3^,^6^,^7^,^8^. Moreover, immune cells and cancer cells interact, and these interactions are affected by the tumor microenvironment. The complex nature of this tumor-immunity-microenvironment interaction favors and sometimes requires a systemic approach in its analysis^2^.

A systemic approach is able to combine quantitative experimental data, mathematical modeling and other methods from computational biology 7. In recent literature, several contributions have made use of this approach to dissect the tumor-immunity interaction^9^,^10^. The interplay between the tumor, the immune system and different types of therapies has been modelled in the last decade^5^,^6^,^8^,^11^, including a study that employed model simulations and patient data to predict the optimal timing and dosage for a therapeutic anticancer vaccination^12^. Although these models in some cases incorporate detailed descriptions of the underlying cell-to-cell communication, they do not take into account the intracellular networks governing immune or tumor cells. Thus, these models by design cannot take advantage of the large amount of omics data produced nowadays to provide molecular-level insights into immunotherapies and their assessment or re-engineering^2^. One option to overcome these limitations is to perform a model-based genotype-phenotype mapping in which model parameters are associated to gene ontology terms^13^. When trying to reconcile simulation results with experimental and clinical data, aggregation of the differentially expressed genes into metagenes will provide a means to connect omics data with model predictions. This is the approach we propose and explore in this paper.

High-throughput data can be combined with mathematical modelling to assess the efficacy of anticancer therapies. For example, Hector *et al*.^14^ quantified apoptosis-regulating proteins in samples of colorectal carcinomas (stage II and III) and normal colonic tissue, and simulated apoptosis signaling to predict the efficacy of apoptosis-inducing therapeutics. Systems approaches have also been used for patient-data-based assessment of experimental immunotherapies. For example, Ulloa-Montoya *et al*.^15^ analyzed biopsy samples from metastatic melanoma patients and identified a pretreatment gene expression signature that can be used to predict the response to immunotherapy.

In this paper we set up and characterized a kinetic model accounting for the interaction of the immune system with melanoma micrometastases as well as for the role of an immunotherapy in controlling and depleting them. It is important to highlight that the work will be focused on the simulation of distributed micrometastases instead of the dynamics of primary melanoma tumors. Here, the immunotherapy refers to treatments stimulating the adaptive immune response, like therapeutic vaccines which are based on melanoma antigens, or patient monocyte-derived dendritic cells loaded with antigens from primary tumor cells^16^.

We combined mathematical analysis, systematic model simulations and statistical techniques to generate phenotypic signatures accounting for melanoma sensitivity and resistance to the inherent immune response and immunotherapy. In the context of this study, the term inherent immune response refers to the patient’s natural immune reactions without any artificial stimulus, while the immunotherapy refers to treatments like dendritic-cell or melanoma antigen vaccination. The phenotypic signatures were compared with metagene signatures derived from clinical data. The comparison not only confirmed the correlation of model predictions with clinical data, but it also allowed the mechanistic interpretation of the clinically derived metagene signatures underlying various biological processes. Furthermore, improvements in the therapeutic anticancer vaccination were proposed based on the model analysis.

Taken together, our results highlight that mathematical models are useful tools for the assessment of tumor immunogenicity, and also that high-throughput data can be employed to detect key genes involved in the tumor-immune system interaction.

## Materials and Methods

### Overview of the methodology

**Figure 1:**
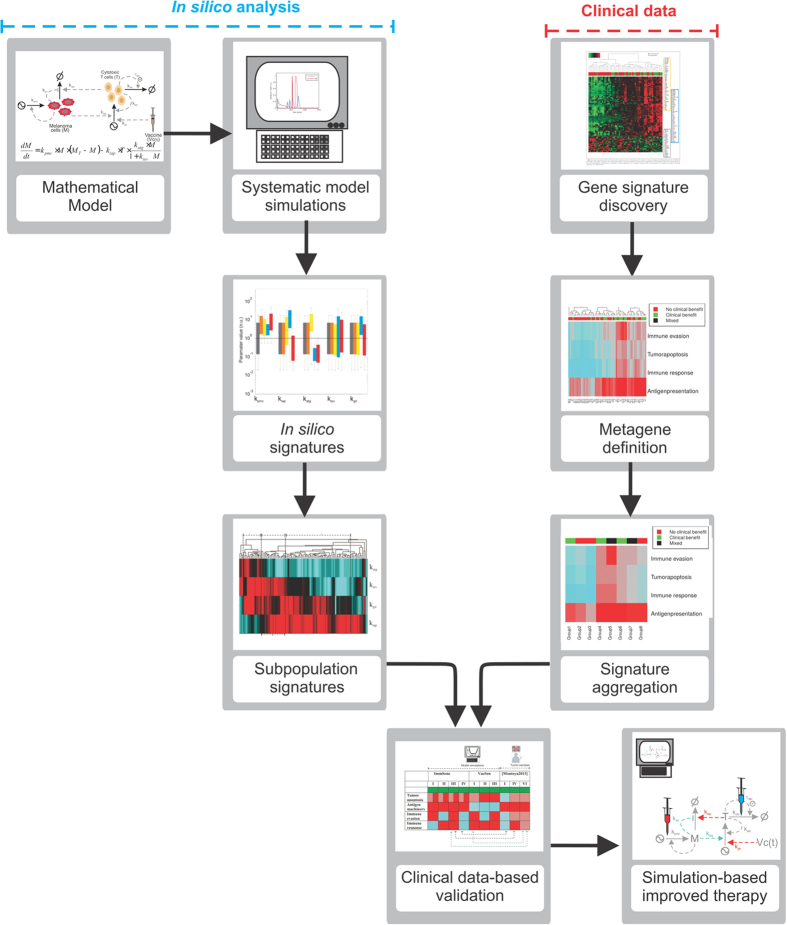
Workflow of the study. Our goal was to generate phenotypic signatures and to compare them with signatures derived from patient data.

The presented method makes use of mathematical modeling to a) infer biological mechanisms explaining gene expression signatures obtained from clinical data and b) propose simulation result -based improvements in existing therapies. The workflow used was as follows (see Fig. 1 for a graphical illustration):

1. A mathematical model describing the biological system under investigation is derived and characterized using published data, and, when possible, model-driven experiments^17^;
2. The model parameters are systematically perturbed and simulations are performed for relevant biomedical scenarios;
3. According to the resulting simulated biological behavior, the model parameter sets are classified into groups. A statistical analysis of the model parameter sets extracted from those groups is used to obtain phenotypic signatures (e.g., patterns in the perturbed model parameter sets for each defined group);
4. Subsequently, clustering analysis is performed to obtain fine-grained signatures of subpopulations within each group. The features of these signatures are linked to the (de)regulation of given biological processes defined in the mathematical model;
5. Mathematical analysis is used to further investigate and define the gene signatures;
6. The genes in clinical signatures are annotated and grouped into metagenes. These metagenes represent genes with a similar gene ontology annotation in terms of the biochemical processes described in the model;
7. The clinical signatures are aggregated using the described metagenes;
8. Phenotypic signatures from the model are compared to metagene signatures from clinical data. Agreement between them allows a biological interpretation of the clinical signatures based on the identification of disrupted or deregulated biological processes. The derivation and calculation of the metagenes is presented in *Gene annotation* and *Metagene grouping* sections;
9. Analysis of additional simulation results are used to propose therapy improvements which must be validated in further experimental and clinical setups.

In the following, the different elements of the procedure are discussed in detail.

### Mathematical model derivation

**Figure 2:**
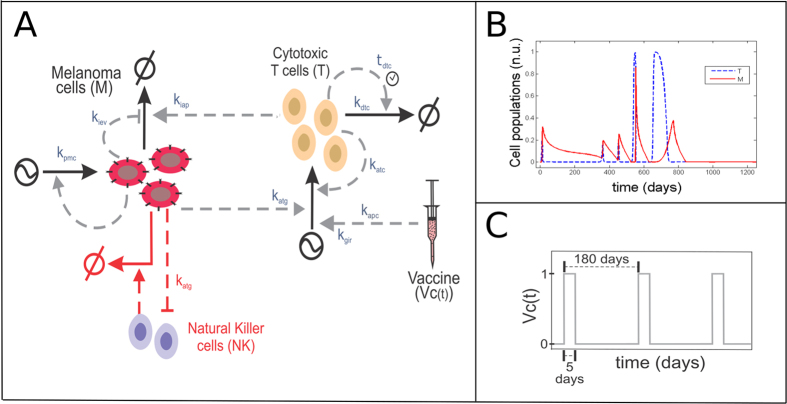
(**A**) Sketch representing the structure of the mathematical model derived to represent the interaction between a melanoma micrometastasis and the natural or therapy-mediated immune response. Parameters: *k*_*pmc*_ – tumor proliferation rate; *k*_*iap*_ – antitumor cytotoxic efficiency; *k*_*atg*_ – antigen presentation efficiency; *k*_*iev*_ – immune evasion efficiency; *k*_*gir*_ – global immune response efficiency; *k*_*dtc*_ – depletion rate of activated cytotoxic T cells (fixed value) *k*_*apc*_ – inherent immune response; t*dtc* – time delay; (**B**) The model can be used to simulate the kinetics of cytotoxic T cells (*T*) in a time-dependent manner and the growth of melanoma cells (*M*) under the corresponding immune stress (*T*(*0*) = *0; M*(*0*) = *0.00081*; nominal values for the model parameters). (**C**) Sketch of the immune therapy simulation used as described in Material and Methods section.

We used published knowledge and preexisting mathematical models describing the interaction between the tumor and the immune system to derive a new simplified kinetic model based on nonlinear ordinary differential equations with time delay^5^,^6^,^18^ (Fig. 2). The model reflects the dynamics of cytotoxic T cells and tumor cells during the growth of melanoma micrometastases, as well as some features associated with the inherent and immunotherapy-induced immune response. It has the following structure:


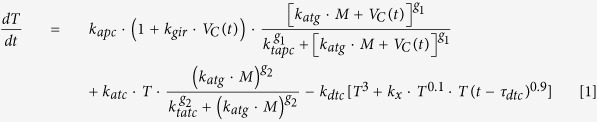



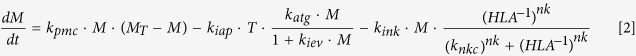


where *T* accounts for the population of cytotoxic T cells and *M* for the population of melanoma cells that compose a micrometastasis. In case of cytotoxic T cells, Equation [1] includes the first term accounting for the activation of cytotoxic T cells by antigen-presenting cells as a result of a) the inherent immune response (*k*_*apc*_) or b) an immunotherapy, for example dendritic cell or antigen vaccination. The immunotherapy-mediated activation of naïve T cells is represented by a time-dependent input variable, *V*_*c*_*(t)* (for details, see “Simulation of vaccine administration” below). An additional feature associated with the immunotherapy is the ability to promote a global unspecific immune response, which amplifies the process of cytotoxic cell activation (*k*_*gir*_).The efficacy of the cytotoxic T cell activation process is dependent on the amount of antigens (*k*_*atg*_) presented by tumor cells (*M*) and follows a saturation dynamics represented by a Hill function with a Michaelis-Menten like parameter (*k*_*tapc*_)^13^ with exponent *g*_*1*_. The equation also includes a term accounting for the self-expansion of activated cytotoxic T cells upon interaction with tumor cells (*k*_*atc*_) which follows a Hill function, with the corresponding Michaelis-Menten like parameter (*k*_*tatc*_) with exponent *g*_*2*_, and is proportional to the amount of antigens (*k*_*atg*_) produced by the tumor cells (*M*). Finally, the equation includes a summand accounting for a biphasic depletion of activated cytotoxic T cells (*k*_*dtc*_). In this term, we included a fast third-order depletion term together with a slower, time-delayed one which accounts for the small fraction of activated cytotoxic T cells that have a longer lifespan (and represent memory T cells in a phenomenological fashion; for time delay estimation and previous kinetic model see^19^). By using this structure for the T cell depletion rate, our model is able to reproduce the basic features of the short-term and memory cytotoxic T cells dynamics with a single differential equation (Supp. Fig. S1).

For the melanoma cells, Equation [2], the first rate term includes a logistic equation accounting for the self-limiting growth of the melanoma cell population (*k*_*pmc*_): we introduced this rate term under the assumption that the melanoma cells in the micrometastasis have not yet broken the blood vessel barrier and therefore achieve a maximum size represented by *M*_*T*_^20^. In addition, the model includes a term accounting for the T cell and NKC-mediated killing of melanoma cells on the second and third term respectively (*k*_*ink*_ ≈ *0.02·k*_*iap*_). In our model, the strength of this process is proportional to the level of antigen presentation (*k*_*atg*_) of the tumor cells *M.* In addition, we also consider the possibility that the melanoma cells could evolve an immune evasion strategy, thereby reducing the efficiency of T cell-mediated recognition and killing (*k*_*iev*_). The final Hill kinetics term, which features the Michaelis like constant *k*_*nkc*_ and the exponent *g*_*nk*_, accounts for the activation of natural killer cells (NKCs) by tumor cells that exhibit low levels of human leukocyte antigen (*HLA*) complexes on the cell surface, and for the ability of activated NKCs to target the tumor cells for killing. *HLA* accounts for the plasma membrane levels of *HLA* complexes, which is assimilated for simplicity into the model parameter *k*_*atg*_ (*HLA* ≈ *k*_*atg*_). This model can be used to simulate the evolution of specific cytotoxic T cell (*T*) populations in a time dependent manner and the behavior of melanoma cells (*M*) under distinct immune-related scenarios (Fig. 2B).

We point out that the derived kinetic model features a combination of mass-action, Hill kinetics and power-law terms, a strategy previously used to model complex regulatory processes in simplified model equations^13^,^21^,^22^. The values of the rate parameters were assigned by surveying published information. For example, the half-life of cytotoxic T cells was used to characterize *k*_*dtc*_. Also, *k*_*tapc*_ and *g*_*1*_ were estimated to induce a sigmoidal APC (antigen presenting cell)-mediated cytotoxic T cell response within the interval of feasible values of the model variables. Alternatively, we reduced some parameters by normalization (e.g., normalizing the expression level of melanoma antigen presentation such that it is equal to 1 in the nominal version of the model). Model parameter values and further explanations are provided in Supplementary Table S1.

### Model simulations

To search for phenotypic signatures accounting for sensitivity or resistance of melanoma cells to inherent and immunotherapy-supported immune responses, we randomly perturbed the values of biologically relevant model parameters using the Latin hypercube sampling method in the logarithmic space. The parameters chosen account for tumor cell proliferation, antigen presentation, efficiency of T cell-mediated killing of melanoma cells, tumor immune escape, and the intensity of systemic immune responses induced by the immunotherapy (respectively *k*_*pmc*_, *k*_*atg*_*, k*_*iap*_, *k*_*iev*_, and *k*_*gir*_). The parameter *k*_*pmc*_ was perturbed in the interval [1, 5]. The upper bound corresponds to with a duplication time of approx. 12 hours for an nominal population M = 0.3. The lower bound was fixed to 1: preliminary simulations indicated that values of *k*_*pmc*_ below initial value did not satisfy the conditions for tumor growth. The parameters *k*_*atg*_*, k*_*iap*_, *k*_*iev*_, and *k*_*gir*_ account for a rather complex aggregation of biological parameters and therefore they were perturbed in the wider interval [0.02 50]. Thereby, we generated 10^4^ solutions, each one with a distinct set of parameter values. For each combination, we performed simulations in three scenarios relevant for describing the interaction between the tumor micrometastasis and the immune system: **scenario 1)** non-immunogenic tumor growth conditions (*M(0)* = *0.00081,* which represents an initiating tumor cell cluster of 30 cells, *T(0)* = *0,* no pre-existing T cell response*, k*_*apc*_ = *0, V*_*c*_*(t)* = *0*); **scenario 2)** inherent antitumor immune response (*M(0)* = *0.00081, T(0)* = *0, k*_*apc*_ = *0.04, V*_*c*_*(t)* = *0*); and **scenario 3)** immunotherapy-supported (vaccine) antitumor immune response (*M(0)* = *0.00081, T(0)* = *0, k*_*apc*_ = *0.04, V*_*c*_*(t)* = *F*_*vac*_*(t*)). Based on exploratory clinical results (data not shown), vaccine administration was simulated by introducing a step-like time-dependent function *F*_*vac*_*(t*) simulated during six subsequent periods of 180 days, with the following structure: *F*_*vac*_*(t)* = *1 if 0* < *t* < *5; F*_*vac*_*(t)* = *0 if 5* < *t* < *180* (see Fig. 2C).

We investigated phenotypic signatures providing resistance to inherent and vaccine-mediated immune responses by simulating the response of the model to the described scenarios. According to the simulation results, the model parameter combinations were classified into the following biologically relevant groups:

- **Tumor**: parameter combinations for which the melanoma cell population is bigger than 90% of the maximum tumor population size (*M* > 0.9 n.u.) after 30 days of non-immunogenic tumor growth conditions (see scenario 1 above). The subsequent groups (b–d) are subclasses of this group;
- **Immune-sensitive**: parameter combinations that generate a growing melanoma micrometastasis in scenario 1, but they are depleted or controlled by the inherent immune response in scenario 2 (*M* < 0.05 n.u. 1500 days after starting of the wild-type immune response);
- **Vaccine-sensitive**: parameter combinations that generate a growing melanoma micrometastasis in scenario 1, are resistant to the inherent immune response in scenario 2, and are depleted or controlled by the immunotherapy-mediated immune response in scenario 3 (*M* < 0.05 n.u. 1500 days after starting the immunotherapy treatment);
- **Vaccine-resistant**: parameter combinations that generate a growing melanoma micrometastasis in scenario 1 and are resistant to both the inherent (scenario 2) and immunotherapy-supported (scenario 3) immune response.

With the criteria chosen, groups b, c, and d do not overlap because our intention was to define “extreme cases”, in a way clear phenotypic signatures could be found. Thus, we are able to unambiguosly assign each solution to one of the groups above. It could be possible to find solutions that do not belong to any of the groups defined, and that may account for intermediate cases (not found in our current results). In this case, the subset of non-allocated solutions maybe further analysed independently.

### Determining phenotypic subpopulation signatures

In order to investigate the potential existence of subpopulations of solutions with a clear-cut distinction in the values of the model parameters, the solutions belonging to each population were examined using hierarchical clustering analysis. The dendrogram and heat map of the solutions were obtained in MATLAB 2014 using the function ‘*clustergram’* which performs hierarchical clustering with the Euclidean distance metric, average linkage and optimal leaf ordering by clustering first along the columns^23^. For visualizing the heat map, we used a customized color map (red for high values and cyan for low values). In the heat map, we classified the solutions in subpopulations that: a) are as large as possible, b) correspond to a division (cluster) in the dendrogram, and c) at least 75 % of the solutions have similar color codes for each model parameter (i.e., the cluster must predominantly be either red or cyan for each model parameter).

### Gene annotation

The 84 genes identified in Ulloa-Montoya *et al*.^15^ were manually annotated with emphasis on classifying them into one of the following categories according to their established role in the immune response: 1) antigen presentation machinery, 2) immune-mediated tumor apoptosis/cytotoxic T cell-mediated killing of tumor cells, 3) tumor immune evasion mechanisms, 4) general immune response and 5) none/unknown. Gene annotation was carried out using the information contained in Ensembl and UniProt databases. In case that the gene function was not or only partially described in context of the phenotypes in question, we searched literature for complementing its annotation using NCBI’s MEDLINE database. The results of the gene annotation procedure can be found in Supplementary Figure S3.

### Metagene definition and estimation

The metagenes defined here represent the averaged expression of the genes that belong to categories 1–4 as defined above. Hierarchical clustering with complete linkage and Euclidean distance was performed on the patient samples using the heatmap.2 function of the gplots package in R. Both margin clusters of the generated heat map had a consistent pattern. These two clusters were used for further analysis. The genes present in the heat map were further assigned to one of the four immune-related metagenes (s. above) and the metagene expression values estimated by taking the arithmetic average of the corresponding genes. Interestingly, we obtained similar values using both arithmetic and geometric mean.

### Comparison between *in silico* and patient signatures

In order to make possible a comparison, the solutions from the *in silico* clusters were log10-transformed and normalized to the interval [−1, 1]. For every cluster, we calculated the average value for each parameter. The average values for the metagenes in the patient clusters of clinical and no clinical benefit were also log10-transformed and normalized to the interval [−1, 1]. We created a matrix by merging *in silico* clusters for vaccine resistant and the two patient signatures and performed hierarchical clustering using the MATLAB 2014 function ‘clustergram’, which performs hierarchical clustering with the Euclidean distance metric, average linkage and optimal leaf ordering by clustering first along the columns. Next, for every *in silico* cluster we calculated the Euclidean distance between its average (X) and the average for the patient clusters (X_Nbn_ and X_Bn_, for non-benefit and benefit respectively) using the following equations:


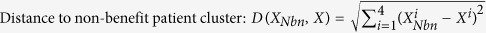



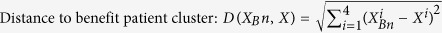


We followed a similar procedure for *in silico* vaccine sensitive clusters. In this case, we also merged into the matrix two representative clusters from the *in silico* immune sensitive solutions.

### Computing of solutions and analysis

Computational simulations and data analysis were performed using MATLAB 2014 running on a Dell Precision T7600 with 2 Intel Xeon E5-2687W 3.1 GHz processors and 64 GB RAM. The computing time for the 10^4^ simulations was in the order of hours.

## Results

### A kinetic model of the interaction between melanoma micrometastasis and the immune system

To investigate the interactions of the tumor with the immune system in the context of melanoma micrometastases and immunotherapy, we set up, characterized and analyzed a kinetic model in nonlinear ordinary differential equations (see Fig. 2). To construct the model, we used published data and mathematical models on the interaction between the tumor and the immune system^5^,^6^,^18^,^24^. The model accounts for the interaction between the local immune system and tumor cells during the growth of melanoma micrometastases. In the model, the variable *M* accounts for the population size of melanoma cells, while *T* accounts for the local population size of specific cytotoxic T cells. The latter one is used as a surrogate for an efficient antitumor response of the adaptive immune system.

Our model for the dynamics of cytotoxic T cells contains terms representing a) the activation of naïve T cells by antigen-presenting cells from the inherent immune system (*k*_*atg*_); b) the self-expansion of activated cytotoxic T cells upon detection of tumor cells (*k*_*atc*_) and c) the depletion of active cytotoxic T cells (*k*_*dtc*_). In addition, the model includes a term for the immunotherapy-mediated activation of naïve T cells, here represented by the time-dependent input model variable *V*_*c*_*(t)*. In our model, the strength of the activation process is modulated by a model parameter (*k*_*gir*_). This parameter accounts for the patient-to-patient variability of the therapy to promote a systemic immune response which amplifies the process of cytotoxic cell activation^15^.

Our model for melanoma cell dynamics contains equations accounting for: a) the growth of the tumor cell population in micrometastases (*k*_*pmc*_); b) killing of melanoma cells by cytotoxic T cells (*k*_*iap*_); and c) biological functions attributed to the antigen presentation machinery by linking its downregulation to the activation of natural killer cells (*k*_*atg*_). In our model, the cytotoxicity of T cells depends positively on the amount of specific antigens presented by the tumor cells, and negatively on the ability of the tumor cells to evade immune response. Both aspects are represented by the tunable parameters *k*_*atg*_ and *k*_*iev*_ (see respectively Parmiani, G. *et al*.^25^ and Umansky, V. & Sevko, A.^26^).

To answer the question which parameter ranges confer immunoresistance to the micrometastasis, we used systematic model simulations to identify regulatory patterns in the tumor-immunity interaction.

### Detection of phenotypic signatures providing immune resistance in melanoma

**Figure 3:**
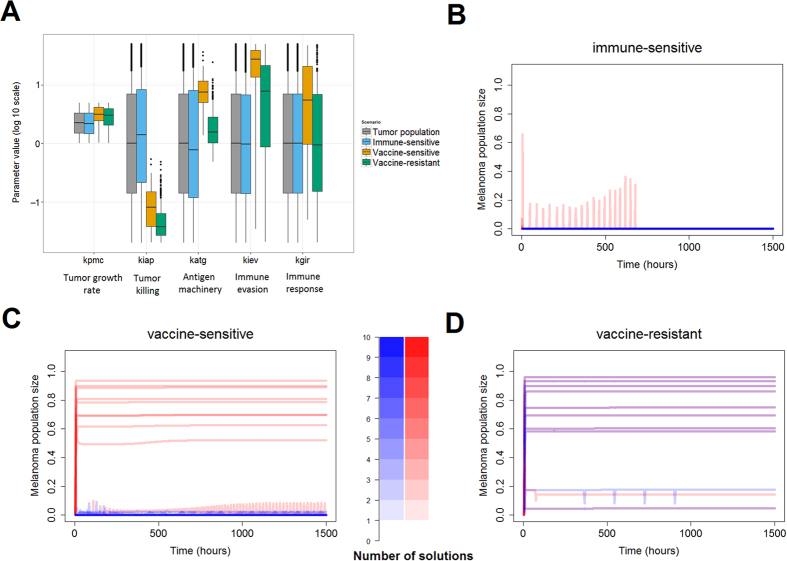
Simulation-result-based detection of signatures responsible for immune resistance or immune sensitivity in melanoma. (**A**) Phenotypic signatures obtained for the four sets of solutions (randomly generated set of parameters). In each boxplot the inner horizontal line is the median, the lower and upper edges are the 25^th^ and 75^th^ percentiles, respectively, and the whiskers extend to the most extreme data points. The ordinate axis origin in the plot accounts for the values in the nominal immune sensitive signature (that is, the model with all the parameters as table S1). Outliers are denoted as dots. See text or caption of Fig. 2 for parameter definitions. (**B–D**) Random **s**ample of 10 solutions from each of the groups immune-sensitive, vaccine-sensitive, and vaccine-resistant, respectively. Red lines correspond to simulation scenario 2 and blue ones to simulation scenario 3. Color bar indicates the number of solutions on overlapping regions.

Recent experimental and clinical data have shown that metastatic melanoma has the ability to evade immunotherapies that are based on antibodies and therapeutic anticancer vaccination^15^,^27^,^28^. In order to detect putative phenotypic signatures promoting immunoresistance of melanoma micrometastases, we generated a set of 10^4^ model parameter configurations through random systematic perturbation. Parameter sensitivity analysis was performed on the solutions to ensure robustness of the simulations (see Figure S4). The solutions of the model parameter combinations were grouped in the following biologically relevant groups (see Fig. 3A and Material and Methods section Detection of phenotypic signatures): i) tumor, those for which the melanoma cell population shows fast growth; ii) immune-sensitive, those combinations for which the inherent immune system can eliminate melanoma micrometastasis; iii) vaccine-sensitive, parameter combinations for which immunotherapy results in effective killing of melanoma cells that were resistant to the inherent immunity; and iv) vaccine-resistant, those model parameter values for which melanoma micrometastasis are resistant to both inherent immunity and to immunotherapy. Fig. 3B–D show model simulations for the scenarios 2 (red) and 3 (blue) in a sample of ten solutions for the groups immune-sensitive, vaccine-sensitive and vaccine-resistant.

According to our simulations, vaccine-sensitivity micrometastases can cope with low T cell-mediated cytotoxicity against tumor cells (*k*_*iap*_) due to the high levels of tumor antigen expression and presentation (*k*_*atg*_) (see Fig. 3A). The results also suggest that the micrometastases, although they express low levels of tumor antigens (*k*_*atg*_), could be sensitive to immunotherapy in case the therapy induces a high systemic immune response and if there is an efficient T cell-mediated cytotoxicity (*k*_*iap*_) (see Fig. 3A). In the case of vaccine-resistant solutions, the genotypic-phenotypic constitution of the tumor in combination with features of the patient’s immune system permits micrometastases to become and remain resistant to both the inherent response and the immunotherapy. Our simulations indicate that lower levels of tumor antigen expression (*k*_*atg*_) in combination with a weak T cell-mediated cytotoxicity (*k*_*iap*_) can render micrometastases refractory to anticancer immunotherapy (see Fig. 3A).

### Elucidation of finer phenotypic signatures using data clustering techniques

**Figure 4:**
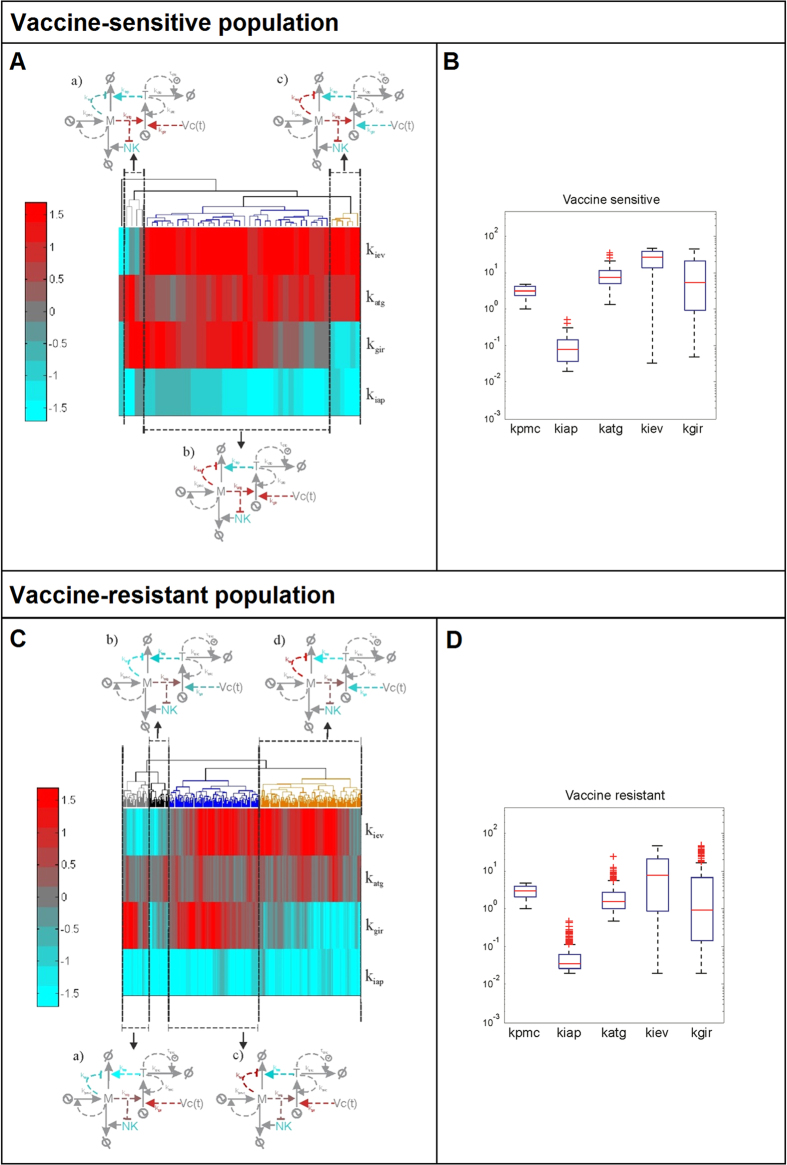
Phenotypic signatures for the subpopulations of vaccine-sensitive (top) and vaccine-resistant (bottom) solutions. Log10 of the nominal parameters in Supplementary Table 1 value is shown (**A,C**). Hierarchical clustering of the solutions in each population with dendrograms and sketches of the regulatory pattern they elicit (**B,D**). Original overall phenotypic signature for each population of solutions.

The previous analysis gave us a rough classification for the model populations. For example, the vaccine-sensitive group extends still across most of the available parameter space accounting for the strength of the immunotherapy-mediated systemic immune response (*k*_*gir*_, Fig. 3A). This revealed the need for additional data analysis techniques to extract subpopulations with a clearer distinction of parameter values. To this end, the solutions belonging to each population were clustered using hierarchical clustering and visualized using heat maps (Fig. 4; see Material and Methods for details). The heat maps were scrutinized to detect finer subpopulations. We here further discuss the results for the vaccine-sensitive and vaccine-resistant solutions, while the analysis of the immune-sensitive solutions is included in the Supplementary Material.

In case of the vaccine-sensitive population, we detected three subpopulations of solutions displaying distinct phenotypic signatures (Fig. 4A,B; subpopulations a, b and c, highlighted with dendrogram lines in grey, blue and orange, respectively). All of them had inefficient T cell-mediated killing of melanoma and high tumor antigen expression and presentation in common (i.e., the value of *k*_*iap*_ is low, and the value of *k*_*atg*_ is high). The differences resulted in two groups:

**group a)** low values of immune evasion (*k*_*iev*_) with high values of immune response (*k*_*gir*_), so a micrometastasis without evasion mechanisms cannot survive in a context of high antigen presentation and vaccination;

**group b/c)** solutions with high values of immune evasion (*k*_*iev*_), either high or low values of immune response (*k*_*gir*_), so high antigen presentation in combination with immunotherapy is enough to counteract the immune evasion mechanisms of the micrometastases. For the purpose of better visualization of the results, every subpopulation is shown connected to a customized illustration of the mathematical model in which the parameters and their associated biological process are colored according to their observed value (Fig. 4A). This allows an understanding of the mechanisms modulating the interactions of the tumor with the immune system in each subpopulation.

In case of the vaccine-resistant solutions we obtained four subpopulations (Fig. 4C,D; subpopulations a, b, c and d, highlighted with dendrogram lines in grey, black, blue and orange, respectively). Our analysis revealed multiple phenotypic signatures, all of them having intermediate levels of antigen presentation in common (*k*_*atg*_):

**group a/b)** solutions with either high or low values of immune response (*k*_*gir*_), low values of immune evasion (*k*_*iev*_) and T cell-mediated killing of melanoma (*k*_*iap*_), meaning that even micrometastases without evasion mechanisms can survive in a context of low cytotoxic effect of T cells and intermediate levels of antigen presentation machinery even under immunotherapy;

**group c/d)** solutions with high value of immune evasion (*k*_*iev*_) with either high or low values of immune response (*k*_*gir*_) and low values of T cell-mediated killing of melanoma (*k*_*iap*_), so the micrometastases survive with high immune evasion mechanisms in the context of intermediate levels of antigen presentation even under immunotherapy.

Taken together, our detailed analysis indicates that each of the three classes (vaccine-sensitive, vaccine-resistant and immune-sensitive, last one presented in Supp. Mat.) can arise through multiple phenotypic signatures.

### Interpretation of phenotypic signatures by mathematical model analysis

In order to further understand the phenotypic signatures, the model was examined to determine the relationship between the parameter values that promote sustained, immunoresistant tumor growth. The condition for tumor resistance and maintenance is the presence of a minimal amount of persistent melanoma cells (existence of a non-zero steady state of *M* and instability on *M* = *0*, see Supp. Mat.). Because the equation for *T* is highly non-linear, a complete definition of the steady state cannot be obtained; instead, we will examine the dynamic behavior of the melanoma cells while the T cell population size remains transiently stable.

Equation [3] represents the parameter dependency that is mathematically defining the condition for a sustained tumor growth (see Supplementary Material for derivation). It considers the relation between the following parameters: the tumor growth rate (*k*_*pmc*_), strength of the T cell mediated killing of melanoma cells (*k*_*iap*_), the level of specific antigens presented by the tumor cells (*k*_*atg*_), the steady amount of T cells (*T*_*s*_), the Hill exponent (*g*_*nk*_) and kinetic parameter of the NKC interaction (*k*_*nkc*_):


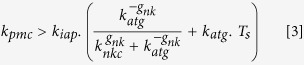


**Figure 5:**
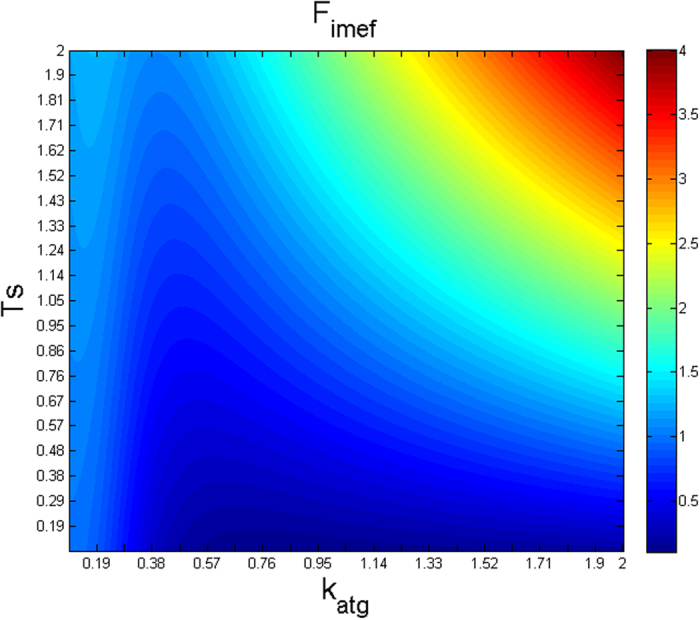
Graphical visualization of the parameter dependency of Equation [3]. The value of the *F*_*imef*_ (showed in the colorbar) is plotted against values of *T*_*s*_ and *k*_*atg*_ (ordinates and abscissas axis ordinate and abscissa, respectively) *F*_*imef*_ corresponds to the term between brackets in Equation [3].

The right part of the inequality can be interpreted as the ability of the patient’s immune system, whether by itself or after therapy, to efficiently counteract micrometastasis growth. Our analysis indicates that when this function is lower than the tumor growth rate (*k*_*pmc*_), the tumor resists the anticancer immunotherapy. In Fig. 5, the factor *F*_*imef*_ is equal to 
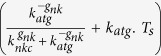
 and accounts for the efficiency of the immune response. Next, for simplicity we considered only the effect of *k*_*atg*_ (level of antigen presentation by the tumor cells) and *T*_*s*_ on the values of *F*_*imef*_ (Fig. 5). The minimum values for *F*_*imef*_ (the blue region in Fig. 5) are reached under intermediate values of *k*_*atg*_ and low values of *T*_*s*_. This blue region corresponds to solutions in which the efficiency of the immune response should be lower than the tumor growth rate (*k*_*pmc*_), so the micrometastasis can resist the immune therapy. This region in which the micrometastasis resists the anticancer immunotherapy can be escaped when the strength of T cell-mediated killing of melanoma cells (*k*_*iap*_) increases, thereby restoring the competence of the patient’s immune system to counteract the tumor. As it was to be expected, for low values of *T*_*s*_, the range in which *F*_*imef*_ has minimum values widens and the patient’s immune system remains unable to suppress tumor growth for a larger region of *k*_*atg*_ values.

In summary, the model-derived analysis indicates that patients who are not competent to eliminate the micrometastasis even in the presence of vaccination (as these results do not depend on the vaccine parameters) are those with a signature of a) low steady-state levels of T cells, which cannot be rescued even by high amounts of antigens presented by the tumor cells (*k*_*atg*_) or b) high levels of T cells with an intermediate level of antigen presentation (*k*_*atg*_). Furthermore, the benefit of the immune response is proportional to the strength of the T cell-mediated killing of melanoma cells (*k*_*iap*_) and inversely correlated with the tumor growth rate (*k*_*pmc*_). These results are consistent with the numerical analysis of vaccine resistance (Fig. 4C, four signatures present intermediate levels of *k*_*atg*_ and low levels of *k*_*iap*_). Furthermore, the equation [3] shows the different combination of parameter values favor tumor cells in vaccine resistance.

### Comparison of mathematical model predictions with molecular data

**Figure 6:**
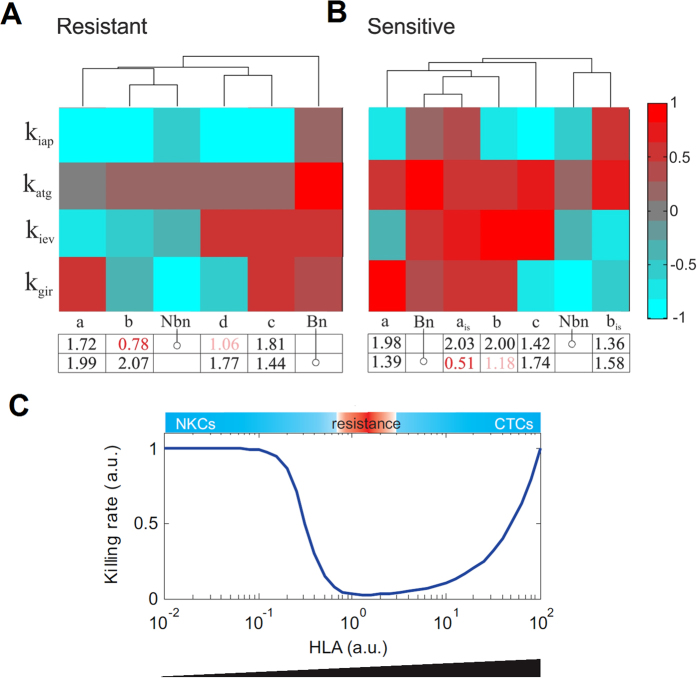
Comparison between the model-based phenotypic signatures and the pretreatment gene expression signature. (**A**) Vaccine-resistant signatures vs. patient clusters. Columns named a, b, c and d correspond to the *in silico* signatures, while columns named Nbn and Bn correspond to the patient clusters without and with clinical benefit, respectively. The phenotypic signatures were clustered using hierarchical clustering (see Material and methods for further details). The table below the heat map displays the Euclidian distances between the average of the *in silico* signatures and the average of the benefit and non-benefit patient clusters. (**B**) Vaccine-sensitive signatures vs. patient clusters. For the purpose of further analysis and discussion, we also included two representative clusters from the in silico immune-sensitive solutions (named *a*_*is*_ and *b*_*is*_) (**C)** Our analysis revealed the need for a tight balance in antigen presentation in the tumor. The total rate of melanoma cell killing (ordinate) was computed and displayed for different expression values of antigen presentation proteins (*k*_*atg*_, abscissa HLA). The total killing rate includes both the contributions of NK and cytotoxic T cells. *k*_*atg*_ ϵ [0.01 100]; *M* = *1*; *T* = *1*, nominal values for other model parameters.

To test our model predictions, we retrieved data from a recently published paper in which biopsy samples of metastatic-melanoma patients were analyzed to define a pretreatment gene expression signature for the prediction of the response to an anticancer immunotherapy^15^. To compare the data with our model predictions, the 84 genes in Ulloa-Montoya *et al*.^15^ were manually annotated and classified into one of the following categories according to their potential role in interactions of the immune system with the tumor: 1) antigen presentation machinery (corresponding to parameter *k*_*atg*_), 2) immune-mediated tumor apoptosis/cytotoxic T cell mediated killing of tumor cells (corresponding to parameter *k*_*iap*_), 3) tumor immune evasion mechanisms (corresponding to parameter *k*_*iev*_), 4) immune response (corresponding to parameter *k*_*gir*_) and 5) none/unknown (see Material and Methods, and Supp. Mat. Table S3). For the categories with productive annotation (1–4), we averaged the expression values of the assigned genes in each patient to come up with one metagene per category. We then aggregated the average relative expression values for these metagenes in clusters of patients with and without clinical benefit (Fig. 6, see also SM figure S3). In order to make possible a comparison, the solutions from the *in silico* and the patient clusters were log10 transformed, normalized and averaged following the procedure explained in Material and methods. We displayed side-by-side the *in silico* phenotypic vaccine-resistant and vaccine-sensitive signatures to make a comparison with the patient groups^15^ and employed hierarchical clustering to generate a heat map grouping *in silico* and patient signatures together (see Material and Methods). As seen in the heat map (Fig. 6), in case of resistant solutions the agreement between the non benefit patient cluster (Nbn) and the *in silico* cluster b is good and is further supported by the small Euclidean distance (highligthed in red). In addition both solutions are qualitatively similar, that is metagenes and parameters keep the same trend of up/down regulation. We notice that, in case of vaccine-resistant solutions, the hierarchical clustering puts aside the benefit patient cluster (Bn). In case of sensitive solutions, none of the three *in silico* clusters conserves the same trend in the up/down regulation of all the parameters compared to the Bn cluster. To further extend our analysis we merged two representative clusters from the *in silico* immune sensitive solutions (a_is_, accounting for immune sensitive solution solutions of high antigen presenting machinery and b_is_ accounting for solutions with low antigen presenting machinery; see Figure S2). Our analysis indicates that cluster a_is_ is similar to the Bn patient cluster. We notice that the hierarchical clustering puts aside the Nbn cluster, clustered together with the b_is_.

Taken together, pairing the phenotypic signatures with those derived from patient data offers a possible means for understanding the biological mechanisms behind the stratification of patients into groups with and without clinical benefit. We think the procedure described can have an application when stratifying patients for therapy or when redesigning immunotherapies.

### Model-predicted co-adjuvants to therapeutic vaccine

**Figure 7:**
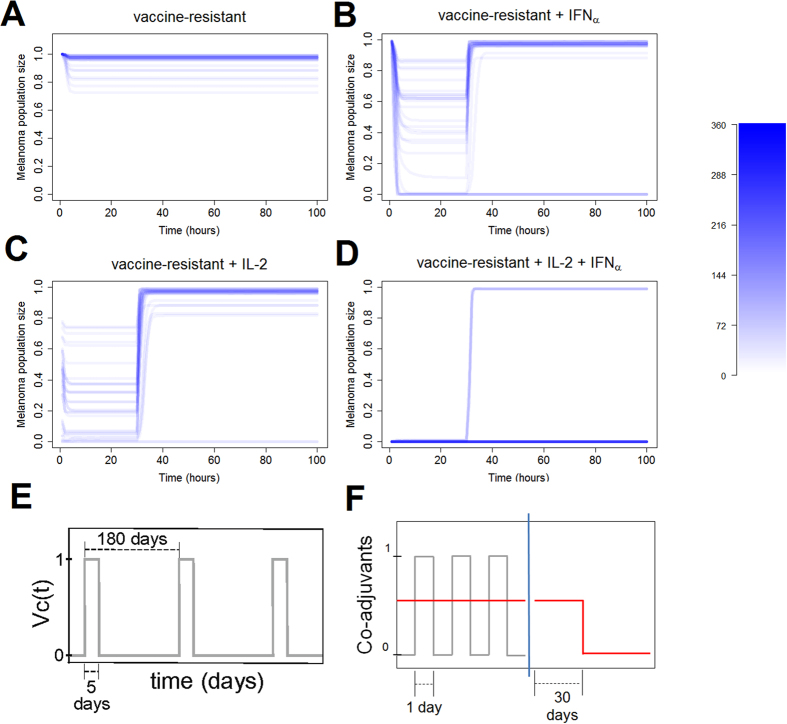
Numerical simulation of the co-adjuvant (IL-2 and IFN-α) therapy. (**A**) A time profile for the vaccine-resistant solutions. (**B**) IFN-α co-adjuvant therapy applied to the solutions from A for 30 days from initial condition. (**C**) IL-2 co-adjuvant therapy applied to the solutions from A for 30 days from initial condition. (**D**) Both co-adjuvants applied in combination to the solutions froms A for 30 days from initial condition. Color bar indicates the number of solutions on overlapping regions. (**E**) Sketch of the immune therapy simulation. (**F**) Sketch of the co-adjuvant therapy simulation (IL-2 and IFN-α), red line is a simplification of the actual dynamics of co-adjuvant therapy, representing the mean value.

We next looked for a way to capitalize on the *in silico* phenotypic signatures obtained in this study. As adjuvant therapies have been proposed and studied in anticancer vaccinations^29^,^30^, we speculated that extension of the model to account for co-adjuvants might provide insights on how to improve the vaccine efficacy in less immunocompetent patients. Based on published results, we decided to explore the combination of immunotherapy with cytokines IL-2 and IFN-α used as co-adjuvants. In our model, we assumed that IL-2 can alter the cytotoxic effect of T cells and T cell proliferation (*k*_*iap*_ and *k*_*atc*_, respectively), while IFN-α increases the presentation of antigens via upregulation of HLAs (*k*_*atg*_). We display simulations of the vaccine-resistant set of solutions either under immunotherapy alone, or in combination with IL-2, IFN-α or both cytokines (Fig. 7). For the treatment with IL-2, the parameters related to T cell proliferation (*k*_*atc*_) and the cytotoxic effect of T cells (*k*_*iap*_) were increased by a factor of 10. For the treatment with INF-α, the parameter for the presentation of antigens (*k*_*atg*_) was increased by a factor of 15. The therapy was simulated during the first 30 days. Afterwards, the simulation was run until day 100. It can be seen that only the combination of both co-adjuvants in conjunction with the immunotherapy is able to deplete the micrometastasis in the high majority of the vaccine-resistant solutions (Fig. 7B,C compared to Fig. 7D).

There is experimental evidence of the benefits of IL-2 or IFN-α supplementation, whether as monotherapy or combined with DC vaccine^29^,^30^,^31^,^32^,^33^. Concerning targeted cytostatic therapies, a cytostatic drug (cetuximab) in combination with vaccination against epidermal growth factor receptor has been shown to be efficacious^34^. Our analysis suggests an improvement of the DC vaccine therapy through the co-administration of both cytokines in patients who do not respond to the vaccine alone, together with targeted cytostatic drugs (reduce *k*_*pmc*_). However, this is an outcome of our modelling exercise and therefore it would in any case require extensive experimental validation.

## Discussion

In this paper, we set up and characterized a kinetic model accounting for the interactions of a tumor with the immune system in the context of melanoma micrometastases. The model reflects the interplay between the inherent immune response, the micrometastases and the anticancer immunotherapies stimulating the adaptive immune response against the tumor. Although the model builds on and is able to mimic many basic features of the tumor-immunity interaction, it is far from being a complete description of the system. Modifications that can improve the model are: a) a more detailed description of the local interactions between the micrometastasis and the immune system, including additional key immune cell populations, cytokines and growth factors which can mediate immune responses and immunosuppression (see models containing this feature in Robertson-Tessi, M. *et al*.^18^ and de Pillis, L. G. *et al*.^6^); and b) a more precise description of the dynamics associated with immunotherapies^11^,^35^, or the addition of clinical data sets^12^. Moreover, the critical signaling and transcriptional pathways controlling the regulation of key phenotypic features in the tumor-immunity interaction could be modelled in detail, providing means for defining more precise gene signatures and hence more accurate model predictions^13^. In line with this, it will be interesting to improve the resolution of the pathways controlling the antigen-mediated dendritic-cell activation and their role in optimizing anticancer vaccination^36^. The model can be expanded to provide more accurate, clinically relevant predictions in accordance with the obtained results. However, the aim of the present work was not to generate a comprehensive model of the interactions of the tumor with the immune system in a melanoma micrometastasis, but to present, describe and illustrate with a case study a methodology that is able to 1) generate phenotypic signatures of the tumor-immunity interaction using kinetic model simulations and mathematical analysis; 2) to compare *in silico* gene signatures with patient data; and 3) to propose model-based co-adjuvants for existing immunotherapies.

We combined systematic model simulations, statistical techniques and mathematical analysis to generate phenotypic signatures accounting for the sensitivity or resistance of melanoma micrometastases to the inherent immune response of individual patients and to the application of immunotherapies. We recently used a similar method to obtain and validate gene signatures of chemoresistance in malignant melanoma^13^, but to the best of our knowledge similar approaches have not been used in the context of interactions of the tumor with the immune system and the assessment of anticancer immunotherapies.

The obtained phenotypic signatures were compared to published data on pretreatment gene expression signatures able to predict the response to immunotherapy in melanoma^15^. To this end, the gene signatures derived from clinical data sets were aggregated by defining and using metagenes which match the features of the biological mechanisms investigated and the mathematical model constructed. This provides a strategy to compare clinical data model predictions, which we think can be applied to other case studies for the assessment of anticancer therapies. Through our analysis, phenotypic signatures identified from model simulations are in agreement with those obtained from patient data sets, but our results also show the existence of additional more fine-grained signatures that can promote immune sensitivity or resistance. This comparison provides support for the interpretation of the predictive signatures obtained in reference 15. Concerning vaccine-resistant signatures, our results and the clinical data indicate that patient immuno-resistant signatures share intermediate (rather than low) expression levels for the genes related to antigen presentation. Our simulations suggest that this tight balance in the expression levels of these genes constitutes a resistance mechanism through which metastatic melanoma cells minimize the effect of immune responses mediated by cytotoxic T cells and natural killer cells, respectively (Fig. 6C). Concerning vaccine-sensitive results, our analysis preferentially grouped the patients with clinical benefit together with an *in silico* signature corresponding to a cluster of immune-sensitive solutions displaying high levels for all the parameters sampled (Figure S2, the cluster a_is_). These results are consistent with previously experimental observations from several cancer immunotherapy trials, in which immunotherapies proved to work in immunocompetent individuals^37^.

Based on the phenotypic signatures, we computationally analyzed the effect of cytokines used as co-adjuvants and proposed potential treatment improvements, in any case require further *in vitro*/*in vivo* experimental validation. The development of a more detailed mathematical model could help improving the predictive and explanatory capabilities of the obtained phenotypic signatures, and hopefully suggest more detailed adjuvant strategies to improve the efficacy of the therapy in patients with a resistant signature. A possible extension of the model could be an adaptation to include the effect of the recently approved immune checkpoint inhibitors^38^ (e.g. anti-CTLA4 and anti-PD1 antibodies) in combination with cellular-based immunotherapies like dendritic cell vaccination.

In the literature, some methodologies have been described to generate regulatory network patterns in the context of biomedicine and biotechnology, which are based on the use of systematic parameter sampling in kinetic model simulations^39^,^40^,^41^. The distinctive features of our methodology are that a) it puts primary emphasis on the detailed definition of the biological scenarios that are simulated and analyzed; and b) we design the model in a way that makes the generated phenotypic signatures comparable with high-throughput data. The ultimate goal of our method is to integrate high-throughput data analysis with model simulations in for the assessment of cancer immunotherapies. Moreover, we think that it will be useful to other immune-related diseases, including infectious ones.

## Additional Information

**How to cite this article**: Santos, G. *et al*. Model-based genotype-phenotype mapping used to investigate gene signatures of immune sensitivity and resistance in melanoma micrometastasis. *Sci. Rep.* **6**, 24967; doi: 10.1038/srep24967 (2016).

## Supplementary Material

Supplementary Information

Supplementary Information
