# Supplementary material for: Model-based genotype-phenotype mapping used to investigate gene signatures of immune sensitivity and resistance in melanoma micrometastasis
Source: Sci Rep. 2016 Apr 26;6:24967. doi: 10.1038/srep24967 (PMC4844979; doi:10.1038/srep24967)
Supplement: Supplementary Information [file srep24967-s1.docx]

# Model-based genotype-phenotype mapping used to investigate gene signatures of immune sensitivity and resistance in melanoma micrometastasis

Guido Santos^1,6^, Svetoslav Nikolov^1,4,5^, Xin Lai^1,2^, Martin Eberhardt^1,2^, Florian S. Dreyer^1,2^, Sushmita Paul^1,2^, Gerold Schuler^2^, Julio Vera^1,2^

**^1^**Laboratory of Systems Tumor Immunology, ^2^Department of Dermatology and University Hospital Erlangen and Faculty of Medicine, Friedrich-Alexander University of Erlangen-Nuremberg, Germany

^4^Institute of Mechanics, Bulgarian Academy of Science, Sofia, Bulgaria

^5^University of Transport, Sofia, Bulgaria

^6^Systems Biology and Mathematical Modelling Group, University of La Laguna, Spain

**Supplementary Material**

**Derivation of analytic gene signature**

For a persistent tumor, the existence of a stable non-zero solution of melanoma cell number (*M*) and instability on its zero solution are prerequisites. First we test the stability of the zero solution. As the steady state for both variables cannot be analytically obtained because of the high non-linearity of the T cell equation (*T*), we will analyze the dynamic behavior of melanoma cells while maintaining T constant (*T_s_*). Under this assumption, the stability of the zero solution of melanoma cells (S.1.1) can be analyzed as the partial derivative of the zero steady state respecting changes on melanoma cells, under the restrictions (S1.2), i.e.

$\frac{dM}{dt}=k_{pmc}.M.\left(M_{T}-M\right)-k_{iap}.T.\frac{k_{atg}.M}{1+k_{iev}.M}-k_{iap}.M.\frac{\left(HLA^{-1}\right)^{gnk}}{\left(k_{nkc}\right)^{gnk}+\left(HLA^{-1}\right)^{gnk}}$ (S1.1)

$M_{T}=1$, $T=T_{s}, HLA=k_{atg}$ (S1.2)

$\left.\frac{\partial\left(\frac{dM}{dt}\right)}{\partial M}\right|_{M=0}=k_{pmc}-\frac{k_{iap}.k_{atg}^{-g_{nk}}}{k_{nkc}^{g_{nk}}+k_{atg}^{-g_{nk}}}-k_{iap}.k_{atg}.T_{s}$ (S1.3)

According to (35), the steady state at $M=0$ is unstable if equation (S1.3) is greater than zero. In (S1.4) and (S1.5) are given these conditions for the instability of the zero solution, i.e.

$k_{pmc}-\frac{k_{iap}.k_{atg}^{-g_{nk}}}{k_{nkc}^{g_{nk}}+k_{atg}^{-g_{nk}}}-k_{iap}.k_{atg}.T_{s}>0,$ (S1.4)

$k_{pmc}>k_{iap}.\left(\frac{k_{atg}^{-g_{nk}}}{k_{nkc}^{g_{nk}}+k_{atg}^{-g_{nk}}}+k_{atg}.T_{s}\right) .$ (S1.5)

Now we will consider the conditions for the existence of stable solutions for *M* different from zero. First, as values of *M* are bounded between 0 and Mt, the solutions for *M* have to be either oscillating or steady for all initial values between these magnitudes. On the other hand, under the assumption that *T* remains constant, *M* cannot oscillate, as the restriction reduces the model to only one variable, so the only possibility for *M* under these conditions is to reach a stable steady state. We are going to consider the solutions which satisfy equation (S1.5), so there is no stable zero solution and, under our reasoning, there must be at least one steady positive solution for *M*. This will be proven now. We solve equation S1.1 under the restrictions (S1.2) and (S2.1)

$\frac{dM}{dt}=0$. (S2.1)

Thus, we obtain equation (S2.2), i.e.

$k_{pmc}.M.\left(1-M\right)-k_{iap}.T_{s}.\frac{k_{atg}.M}{1+k_{iev}.M}-k_{iap}.M.\frac{k_{atg}^{-g_{nk}}}{k_{nkc}^{g_{nk}}+k_{atg}^{-g_{nk}}}=0 .$ (S2.2)

This equation can be solved analytically, and we obtain three different solutions, one of them is the zero solution, which is going to be instable under assumption (S1.5). Of solutions$\bar{M}_{2,3}$,at least one must be positive for all values of the parameters satisfying S2.2.

$\bar{M}_{1}=0,$ (S3.1)

$\bar{M}_{2,3}=\frac{k_{pmc}.k_{iev}-k_{pmc}-A\pm\sqrt{\left(k_{pmc}.k_{iev}-k_{pmc}-A\right)^{2}+4k_{pmc}.k_{iev}\left(A-k_{iap}.k_{atg}.T_{s}\right)}}{2.k_{pmc}.k_{iev}}$ (S3.2)

where $A=\frac{k_{iap}.k_{iev}.k_{atg}^{-g_{nk}}}{k_{nkc}^{g_{nk}}+k_{atg}^{-g_{nk}}}$.

Applying condition (S1.5) for (S3.2) makes the right term inside the root positive. Because the left term in the root is equal to the one in front of the root before squaring, the root itself will have a higher value than the term in front of it. That means that the sign of the solutions will be the sign of the root term. In this condition, there is going to be one positive and one negative solution, so there is at least one positive solution that will always exist when the zero steady solution is instable.

Summing up, when equation (S1.5) is satisfied, at least one positive solution for *M* exists and the zero steady solution will be instable.

**Figure S1. Biphasic depletion of active cytotoxic T cells**

| 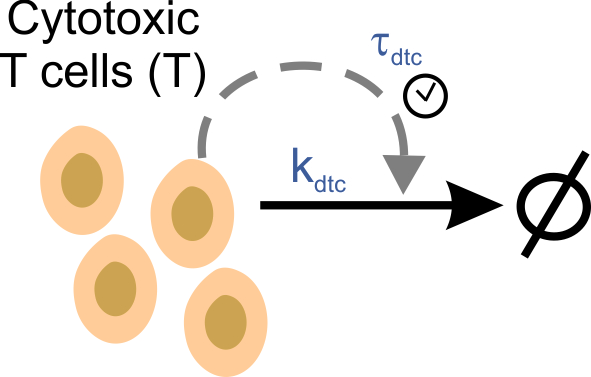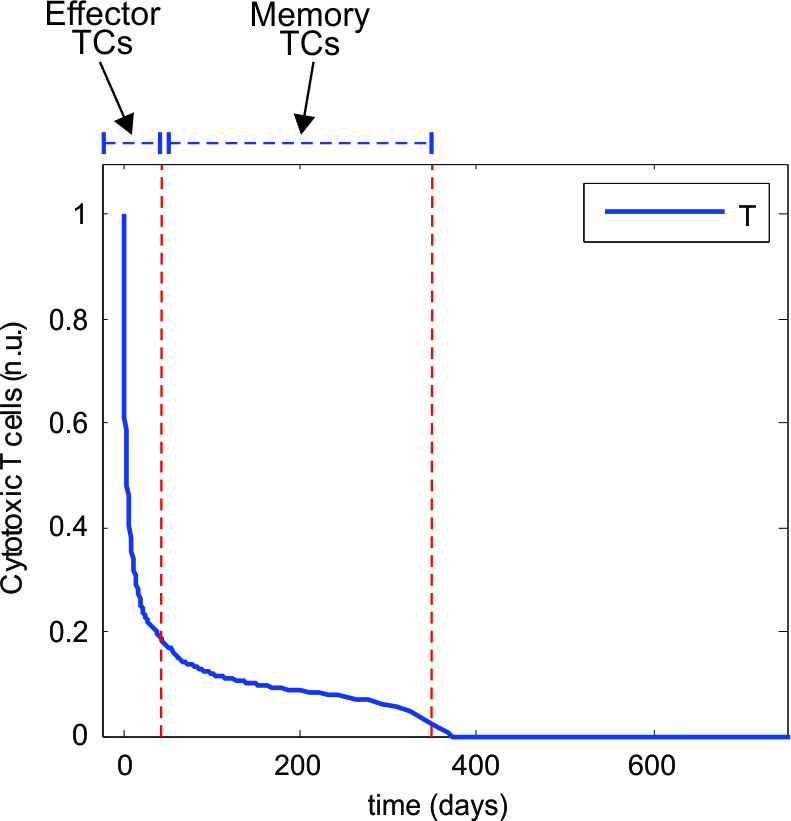 |
| --- |

**Figure S1. Biphasic depletion of active cytotoxic T cells.** Upon the introduction of a slower, time-delayed T cell depletion term, our model can account for a small fraction of activated cytotoxic T cells (approx. 10 %) that have a longer lifespan and act as a phenomenological representation of memory T cells [19]. (*T(0)=1; M(0)= 0*; nominal values for the model parameters ).

**Table S1. Model parameter definition and values**

| **Parameter** | **Description of associated process** | **Nominal Value** | **Reference and Comments** |
| --- | --- | --- | --- |
| *k_apc_* | APC-mediated initiation of cytotoxic T cell response | 0.04 day^-1^ | PMID:9182685 |
| *k_gir_* | Vaccine mediated global immune response | 1 a.u. | Normalized |
| *k_atg_* | Expression of melanoma specific antigen | 1 a.u. | Normalized |
| *k_tapc_* | Threshold APC-mediated initiation of cytotoxic T cell response | 0.2605 a.u. | Fitted to normalization^†^ |
| *g_1_* | Hill-coefficient DC-mediated initiation of cytotoxic T cell response | 2.784 | Fitted to normalization^†^ |
| *k_atc_* | Expansion of active cytotoxic T cells | 2.0791 day^-1^ | PMID:14530309 |
| *k_tatc_* | Threshold expansion of active cytotoxic T cells | 0.2605 a.u. | Fitted to normalization^£^ |
| *g_2_* | Hill-coefficient expansion of active cytotoxic T cells | 2.784 | Fitted to normalization^£^ |
| *k_dtc_* | Main (linear) depletion of effector cytotoxic T cells | 0.3466 day^-1^ | PMID:14530309 |
| *k_x_* | Fraction of memory T cells | 0.1 a.u | PMID:14530309 |
| *τ_dtc_* | Half-life of memory T cells | 500 days | PMID:14530309 |
| *k_pmc_* | Proliferation of melanoma cells | 0.5545 day^-1^ | PMID:20406486 |
| *k_iap_* | Cytotoxic T cell-mediated melanoma cell apoptosis | 5.4931 day^-1^ | PMID: 15725959 |
| *M_T_* | Maximum size of micrometastasis | 1 a.u. | Normalized^¥^ |
| *k_iev_* | Melanoma-elicited immune evasion mechanism | 1 | Normalized |
| *M_0_* | Initial condition melanoma micrometastasis | 0.00081 a.u. | Normalized^¥^ |
| *g_nk_* | Hill exponent for NKC interaction | 3.9913 | Fitted to normalization^†^ |
| *k_nkc_* | Kinetic parameter for NKC interaction | 3.1623 | Fitted to normalization^†^ |

Notes to the table: ^†^ .The values of Hill equation coefficients were calculated such that the APC-mediated initiation of cytotoxic T cell response reaches 95 % of the saturation value for a tumor population size of 75 % of the maximum micrometastasis size and normal expression level of melanoma specific antigens. A similar assumption was used to calculate^£^. ^¥^. PMID:12794026, PMID:16968875, PMC:2922988

**Figure S2. *In silico* signatures for immune-sensitive subpopulations from the model. A.** Hierarchical clustering of the solutions in each population, with dendrograms. **B.** Original overall phenotypic signature for the each population of solutions.

|  | | |
| --- | --- | --- |
| **Immune-sensitive population** | | |
| **A**  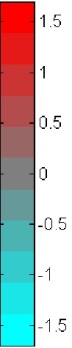 | **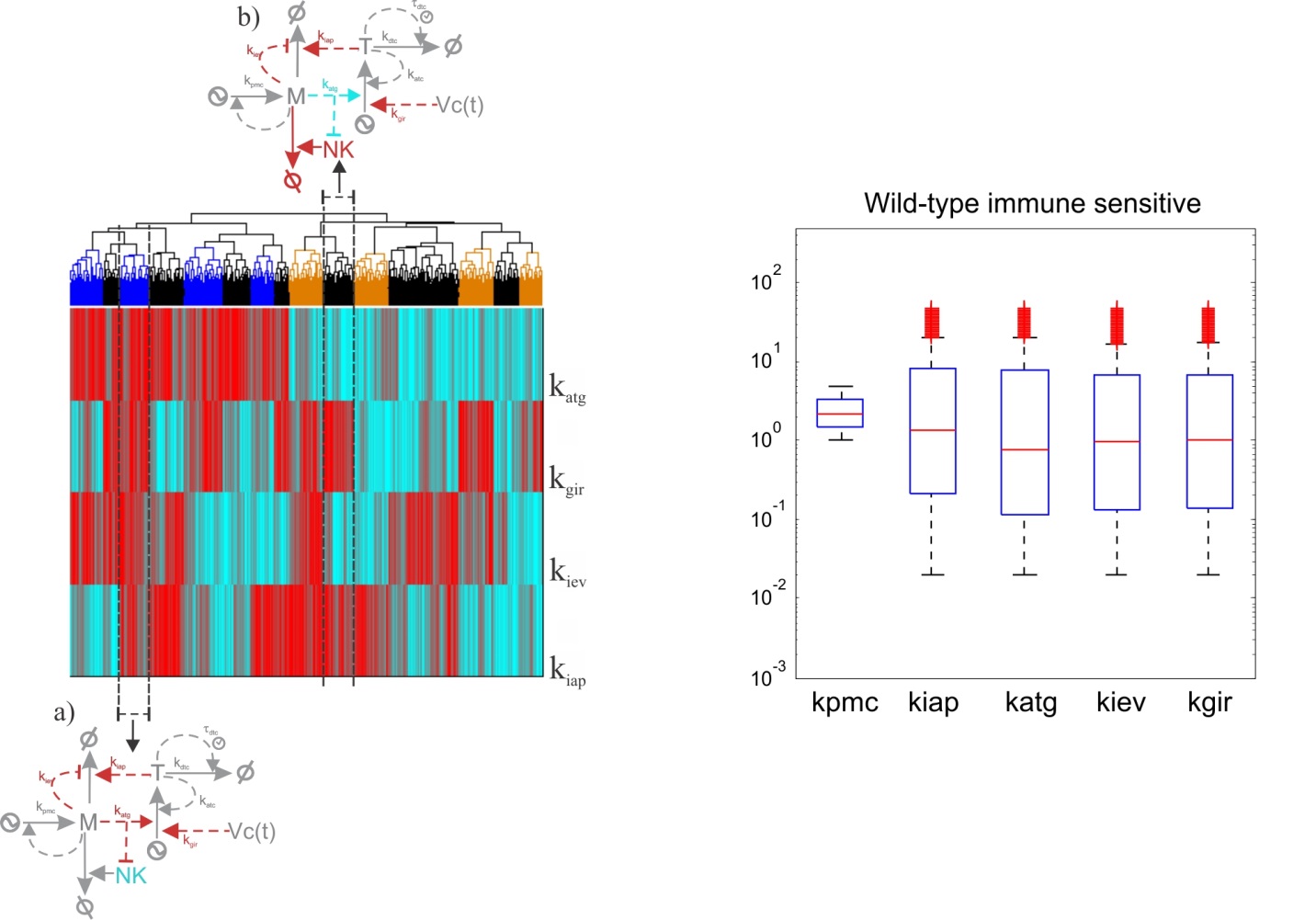** | **B**  **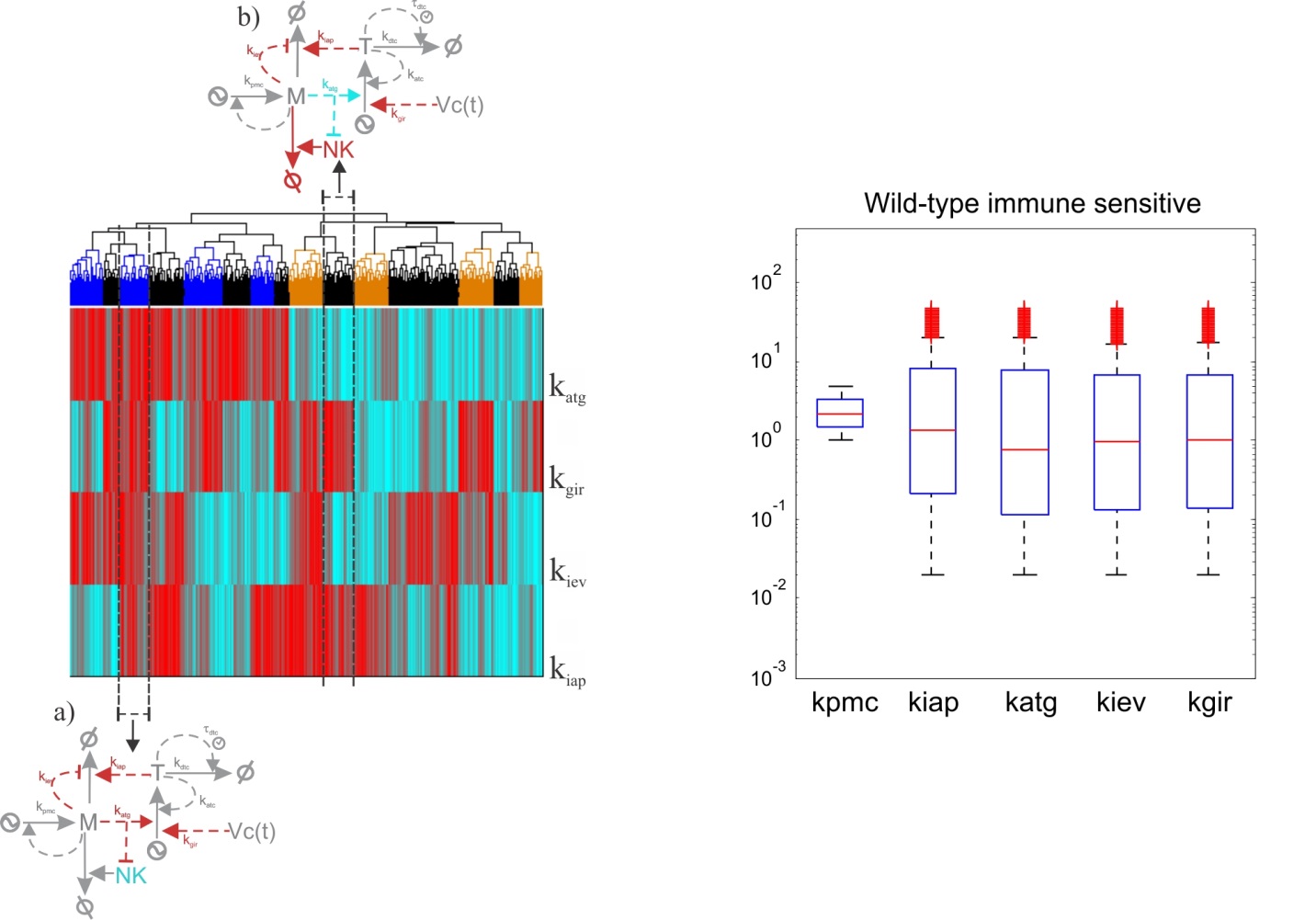** |

**Figure S3. Grouping of patient data into clusters of clinical benefit vs. no clinical benefit.**

| Original patient clustering | Clustering after gene and patient aggregation |
| --- | --- |
| 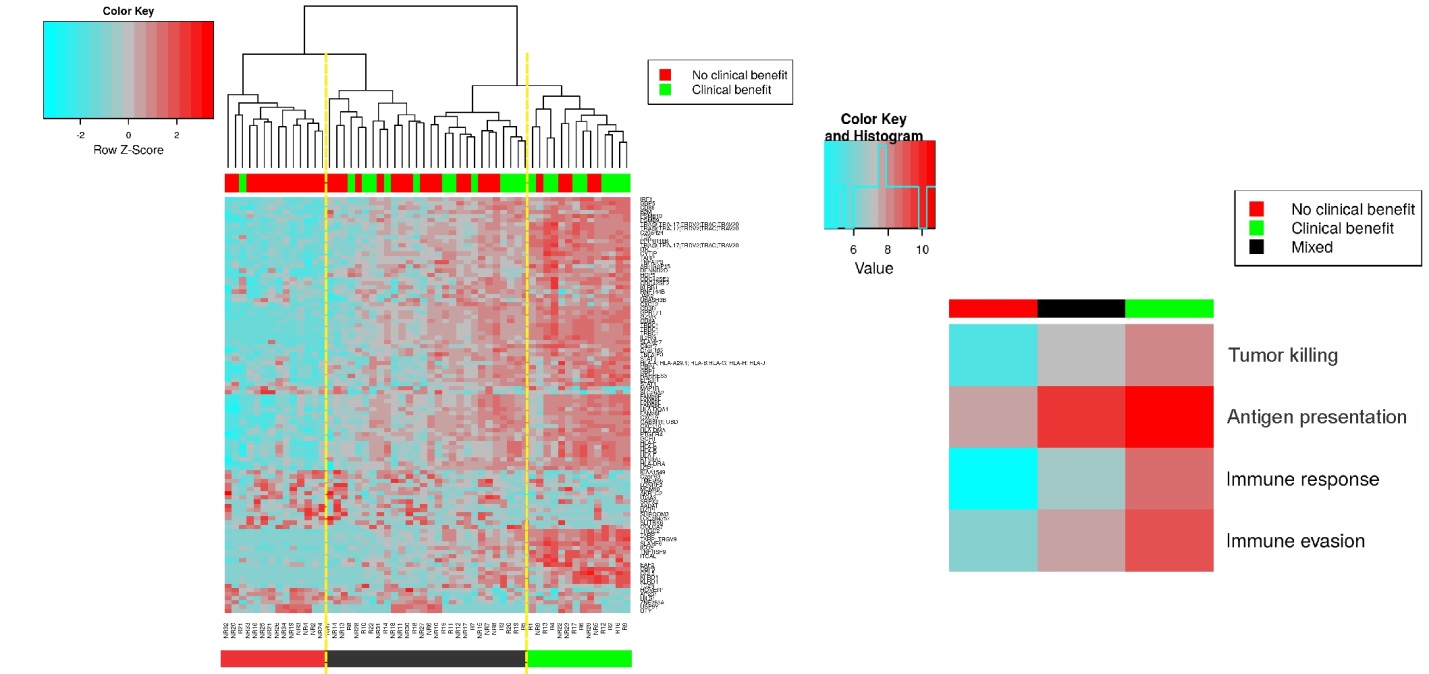 | |

**Figure S4. Sensitivity analysis for the four groups of solutions: tumor, immune-sensitive, vaccine-sensitive and vaccine-resistant.** The sensitivities are calculated dynamically, they measure the ratio of the log change in the integral of the simulation of the variables over the log change in the parameters. The panels show histograms for the mean value of parameter sensitivities of each solution. The vast majority of the solutions have a mean value of sensitivity less than one, which means that changes in parameters affects very little to the dynamical behavior of the solutions.


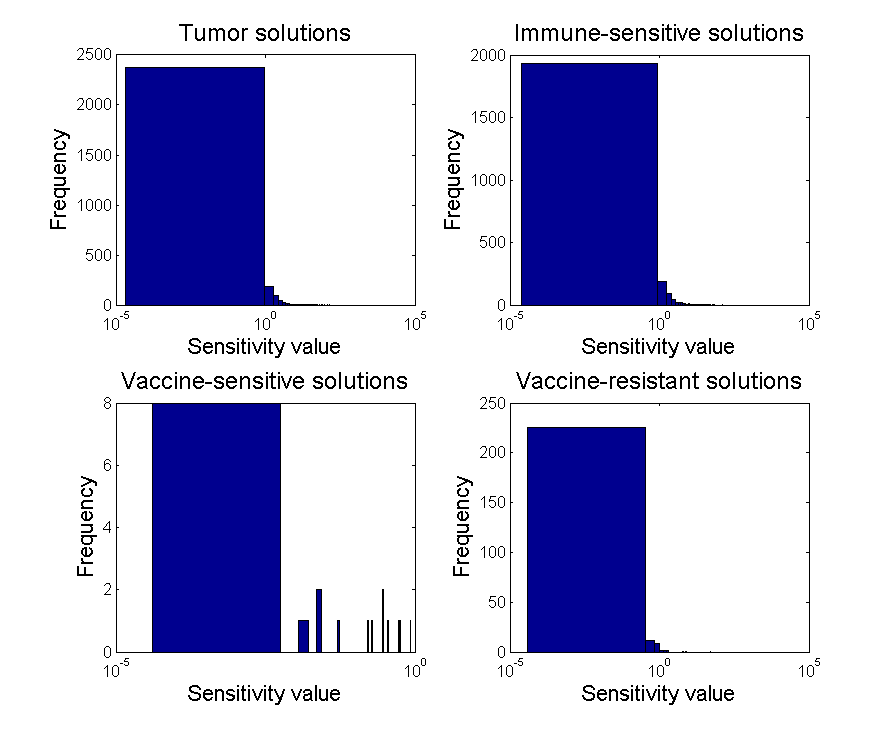


**Table S2. Numerical data of the Figure 7 in the text.** Data are normalized between 0 and 1.

| Resistant | a | b | Nbn | d | c | Bn |
| --- | --- | --- | --- | --- | --- | --- |
| k_iap_ | -0.97 | -1.00 | -0.50 | -0.89 | -0.91 | 0.30 |
| k_atg_ | 0.05 | 0.11 | 0.20 | 0.17 | 0.17 | 1.00 |
| k_iev_ | -0.71 | -0.54 | -0.30 | 0.63 | 0.61 | 0.50 |
| k_gir_ | 0.60 | -0.42 | -1.00 | -0.59 | 0.53 | 0.50 |

| Sensitive | a | Bn | a_is_ | b | c | Nbn | b_is_ |
| --- | --- | --- | --- | --- | --- | --- | --- |
| k_iap_ | -0.65 | 0.30 | 0.41 | -0.74 | -0.83 | -0.50 | 0.53 |
| k_atg_ | 0.60 | 1.00 | 0.54 | 0.55 | 0.66 | 0.20 | 0.66 |
| k_iev_ | -0.33 | 0.50 | 0.67 | 0.93 | 1.00 | -0.30 | -0.70 |
| k_gir_ | 0.93 | 0.50 | 0.48 | 0.53 | -0.76 | -1.00 | -0.47 |
